# Remote refocusing for multi-scale imaging

**DOI:** 10.1117/1.JBO.29.8.080501

**Published:** 2024-08-08

**Authors:** Md Nasful Huda Prince, Nikhil Sain, Tonmoy Chakraborty

**Affiliations:** a University of New Mexico, Department of Physics and Astronomy, Albuquerque, New Mexico, United States; b University of New Mexico, Comprehensive Cancer Center, Albuquerque, New Mexico, United States

**Keywords:** microscopy, remote focusing, volumetric imaging, multi-scale imaging

## Abstract

**Significance:**

The technique of remote focusing (RF) has attracted considerable attention among microscopists due to its ability to quickly adjust focus across different planes, thus facilitating quicker volumetric imaging. However, the difficulty in changing objectives to align with a matching objective in a remote setting while upholding key requirements remains a challenge.

**Aim:**

We aim to propose a customized yet straightforward technique to align multiple objectives with a remote objective, employing an identical set of optical elements to ensure meeting the criteria of remote focusing.

**Approach:**

We propose a simple optical approach for aligning multiple objectives with a singular remote objective to achieve a perfect imaging system. This method utilizes readily accessible, commercial optical components to meet the fundamental requirements of remote focusing.

**Results:**

Our experimental observations indicate that the proposed RF technique offers at least comparable, if not superior, performance over a significant axial depth compared with the conventional RF technique based on commercial lenses while offering the flexibility to switch the objective for multi-scale imaging.

**Conclusions:**

The proposed technique addresses various microscopy challenges, particularly in the realm of multi-resolution imaging. We have experimentally demonstrated the efficacy of this technique by capturing images of focal volumes generated by two distinct objectives in a water medium.

## 1 Introduction

In the realm of biological research, obtaining comprehensive 3D volumetric images is crucial for gaining insights into the functionality of specimens. This process entails capturing multiple 2D cross-sectional slices at varying depths, demanding precise optical sectioning and focus. However, achieving deep imaging while minimizing aberrations and ensuring fast acquisitions of these 2D slices is a challenging task. To address these challenges, various methodologies have been developed to maintain specimen integrity and stabilize the objective. One of the most significant advancements in the last decade, proposed by Botcherby et al.,^1^^,^^2^ is the remote focusing method, which employs two pupil-matched objectives to carry out remote focusing (RF). This technique demonstrates its potential to image sample depths while effectively mitigating aberrations, specifically for high numerical aperture (NA) systems. What sets this method apart is its remarkable ability to map the entire volume from one medium to another instantaneously and then sample at a volumetric speed limited solely by the camera’s capabilities.^3^^,^^4^

Due to this key feature, RF has been widely used for constructing different types of microscopy systems and imaging a broad spectrum of biological samples. Its applications are particularly notable in the development of advanced systems such as spinning disc^5^ and multiphoton microscopy.^6^ In axially swept light-sheet microscopy (ASLM), RF is utilized to move the light-sheet across the field of view using mechanical actuators placed remotely.^7^^,^^8^ This method allows microscopes to cover nearly a square millimeter field of view while keeping the resolution uniform.^9^ In addition, RF is crucial in operating specific microscopes such as the oblique plane microscope, for which it aids in imaging a tilted beam.^10^^,^^11^ Beyond these, RF is now being used in the detection part of microscopes for creating fast, volumetric image stacks.^4^ Its significant influence on imaging has led to numerous studies focusing on the alignment sensitivity of RF^12^ and the various aberrations that it may introduce.^13^

**Fig. 1:**
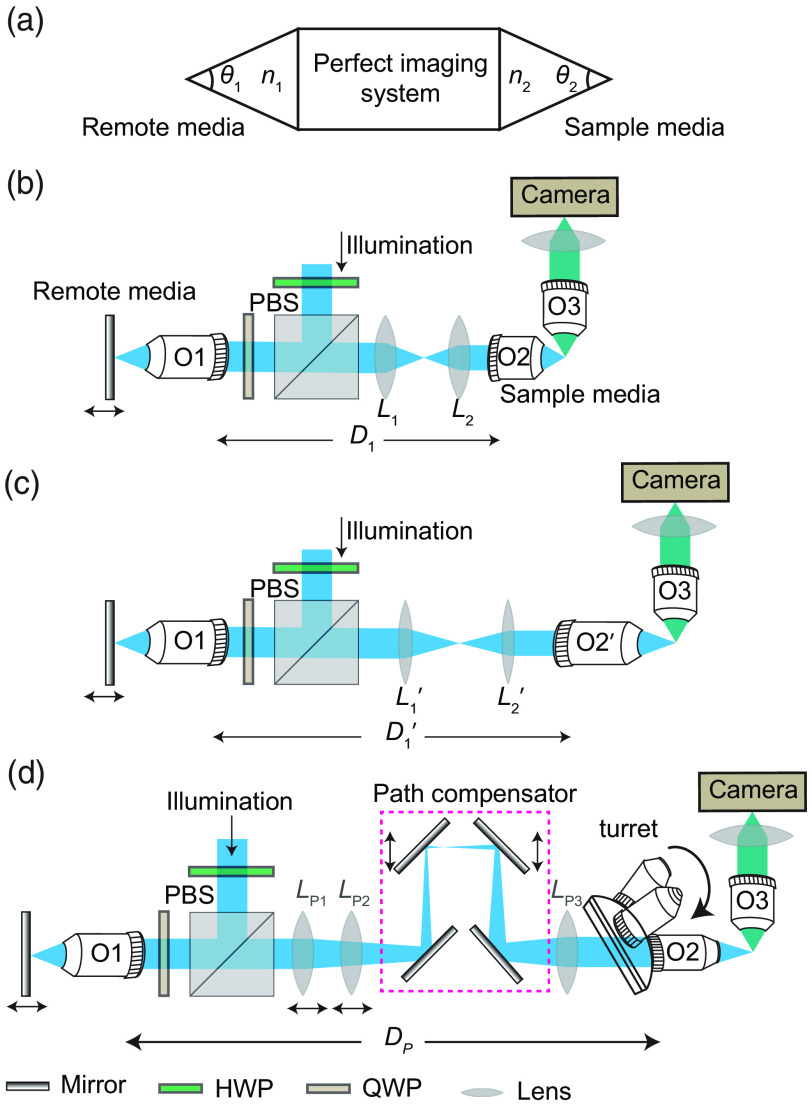
Schematic diagram of the RF system. (a) The RF-based perfect imaging system that requires an equal aperture angle ($\Theta_{1}=\Theta_{2}$) in two different media with RIs of $n_{1}$ and $n_{2}$. (b) Typical RF technique employing a commercially available lens pair ($L_{1}$ and $L_{2}$) to match objectives O1 and O2 with a compromise of magnification matching. The 4f configuration demands a path length ($D_{1}$) between them. (c) Switching the objective ($O2^{\prime }$) necessitates replacing of the lens pair by $L_{1^{\prime }}$ and $L_{2^{\prime }}$ and adjusting the optical path length to $D_{1^{\prime }}$ in between the objectives. (d) The proposed customized optical design aims to achieve perfect magnification matching by creating a customized focal length lens, maintaining 4f configuration by compensating for the light path length for different objectives, and offering the flexibility of multiple resolution options by seamlessly switching between objectives.

With that said, the implementation of RF systems necessitates adherence to three crucial criteria, often imposing limitations on the mechanical design and the off-the-shelf optics. The first requirement, referred to as the resolution requirement, hinges on the pair of objectives selected for RF. In the ideal imaging system within an RF setup, equal aperture angles in both the sample and remote media are essential ($\Theta_{1}=\Theta_{2}$), as depicted in Fig. 1(a). This will only be satisfied if the angular aperture of the remote space objective (O1), called the remote objective hereafter, becomes at least equal to the angular aperture of the sample space objective (O2) (Fig. S1 in the Supplementary Material). This is a condition for resolution preservation in RF, and this condition places a restriction on the maximum NA that an objective can possess for optimal imaging. The determining factor for this maximum NA is the ratio of the refractive index (RI) of the sample to that of the remote media ($n_{2}/n_{1}$).

The second critical requirement for the RF is ensuring that the magnification of the objective associated with the sample media (O2) matches that of the remote media (O1), and this match should be in accordance with the RI ratio, as illustrated in Fig. 1(b). Typically, the overall magnification of the sample media should correspond to the refractive index (RI) ratio of the remote media to the sample media. Failure to meet this stringent requirement results in a suboptimal performance in terms of residual aberrations and the diffraction-limited range. For instance, a recent study by Mohanan and Corbett^14^ suggests that even a 1% magnification mismatch for high NA objectives may lead to a 50% reduction in the diffraction-limited range. Achieving such magnification-matching necessitates a specialized lens pair ($L_{1}$ and $L_{2}$) to conjugate the pupils of the two objectives (O1 and O2). However, the exact lens pair is often not readily available in the manufacturer’s lens catalog. Recently, efforts have been made to implement RF by introducing an automatic customized tube lens.^15^ In addition, in a separate endeavor, a customized zoom lens was developed to rectify the magnification mismatches caused by various sample dipping media with different RIs.^16^

The third requirement is that the RF technique mandates the maintenance of a 4f design configuration to correct the spherical aberration for the out-of-focus plane of the remote media. However, it is important to be careful when arranging such a system to make sure the tube lenses form a correct 4f imaging setup, accurately linking the pupil planes. As mentioned by Botcherby et al.,^1^ although it is a routine practice in microscopy that the infinity-corrected tube lenses are often placed closer to or farther from the objective than what their intended position was designed for, in RF systems, any extra phase curvature caused by points displaced along the axis could lead to a decline in the system’s performance.

Such stringent optical requirements often impose limitations on the use of RF-based techniques in microscopy, particularly in applications requiring flexibility, such as changing objectives in a multi-resolution microscope. For instance, when switching from one sample-dipping objective (O2) to another objective ($O2^{\prime }$) with different back pupil sizes, focal lengths, magnifications, and NAs, a new lens pair ($L_{1^{\prime }}$ and $L_{2^{\prime }}$) is needed to carry out the pupil matching with the remote media (O1), as depicted in Fig. 1(c). This further complicates the process because such a change necessitates a different path length ($D_{1^{\prime }}$) to preserve the 4f configuration, unlike the path length required for O2, as shown in Fig. 1(b). Achieving a multi-resolution microscope modality or altering the aberration-limited imaging depth within the sample demands the ability to switch objectives without affecting existing optical elements, specifically the RF objective, the matching lens pairs, and their relative distances.

In this work, we introduce an innovative optical technique designed to streamline the formation of a customized lens pair and a path compensator for executing the RF. This method ensures precise magnification matching, upholding a 4f optical configuration. In addition, it facilitates multi-resolution imaging simply by rotating a turret, adjusting the linear positions for two lenses ($L_{P1}$ and $L_{P2}$) and two mirrors, and positioning them at pre-calibrated locations, as illustrated in Fig. 1(d). The method is empirically validated through the interchange of two multi-immersion objectives in water, each having distinct focal lengths and NAs. Remarkably, this customized setup not only matches but, in some cases, surpasses the performance of traditional RF systems. By carefully selecting the remote objective and lenses, particularly their diameter and focal lengths, this technique significantly increases the ease and flexibility of transitioning between various objectives. In addition, our method can seamlessly incorporate the concept of any immersion remote refocus (AIRR) microscopy and employ the dynamic zoom tube lens^16^ to not only correct for the RI mismatch but also accommodate multiresolution objectives.

## 2 Methods

Our method to accommodate multiresolution microscopy through a single RF geometry starts by identifying the objective for the remote media. As described above, because a perfect imaging system in an RF setup relies on maintaining an equal angular aperture in the two media, ($\Theta_{1}=\Theta_{2}$) in Fig. 1(a), the choice of RF objective (O1) sets a limit on the maximum numerical aperture (NA) of the sample space objective (O2) that can be employed to carry out multiresolution imaging. When doing this for multiple objectives with different NAs, such as in our case, one must identify the highest NA objective from the list and use that to select the O1 by multiplying the RI ratios ($n_{1}/n_{2}$) to that of the NA of O1.

The next step is to identify the matching lenses so that the overall magnification of the RF setup comes out to be $n_{2}/n_{1}$. This means that, for a traditional RF geometry, one will have to identify different sets of lens pairs [$L_{1}/L_{2}$ in Fig. 1(b) and $L_{1^{\prime }}/L_{2^{\prime }}$ in Fig. 1(c)] for each sample space objective. Inspired by the capabilities of the automatic tube lens^15^ and the customized zoom lens design,^16^ here, we propose using three lenses instead of two to carry out the pupil matching [Fig. 1(d)]. Our approach involves fixing one lens ($L_{P3}$) while carefully selecting the focal lengths and their relative distances between the two other lenses ($L_{P1}$ and $L_{P2}$) to recreate the varied lens pair ratio requirement. The distance between these lenses can then be adjusted using a motorized linear stage to pre-determined positions, thereby forming customized focal lengths, which are essential for matching different objectives. By utilizing the paraxial approximation, we traced rays and established customized focal lengths using identical lens pairs ($f_{\mathrm{LP}1}$ and $f_{\mathrm{LP}2}$) to match two distinct objectives (O2 and $O2^{\prime }$).^17^ The only variation between each case lies in the distance between them, resulting in a customized focal length measured from the corresponding principal plane (Fig. S2 and Note 1 in the Supplementary Material).

The final step is to ensure that the 4f geometry relaying the sample and the remote objective is maintained. For this, we introduced a path compensator, depicted in the magenta square box in Fig. 1(d), which ensures that the required distances between the remote objective and the sample objectives ($O2/{O2}^{\prime }$) are always at the correct position as warranted by their matching lens pair. The path compensator comprises four mirrors in a beam folding configuration: two are held in a fixed position and two are mounted on a linear translation stage. Depending on the optical path length requirement (D1 and $D1^{\prime }$ in Fig. 1), the linear stage is translated to a predetermined position to ensure that 4f geometry for both the O1 to O2 and O1 to $O2^{\prime }$ configurations are met precisely.

**Fig. 2:**
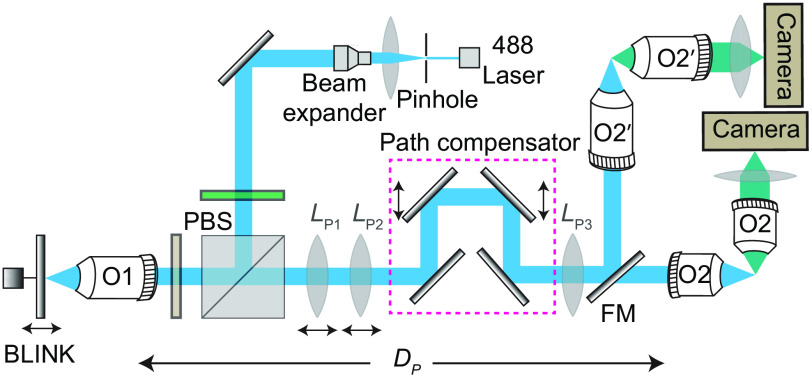
Schematic showing the experimental setup. The setup allows us to alter the optical path by putting the flip mirror (FM) before the illumination objective. The customized RF technique ensures the perfect matching of two different objectives (O2 and O2’) with a remote objective (O1) using identical optical elements while preserving the requirement for the perfect imaging system. We used identical illumination and detection objectives to assess the performance of the customized RF system. FM, flip mirror.

We tested the working of our proposed idea by building two separate remote focusing setups, each equipped with two pairs of illumination and detection objectives (Fig. 2) such that both can be controlled by one remote focusing unit. Our illumination arm comprises a linearly polarized 488-nm laser beam (Coherent Sapphire, Daventry, United Kingdom), which was focused through a 50-mm achromatic doublet (AC254-50-A, Thorlabs, Newton, New Jersey, United States) onto a 30-μm pinhole (P30D, Thorlabs). The beam was then recollimated using a 200-mm achromatic doublet (AC254-200-A-ML, Thorlabs). A 5× Galilean beam expander (GEB05-A, Thorlabs) expands the original beams by 20 folds before being fed into the remote objective O1 through a polarizing beam splitter (10FC16PB.7, Newport, Irvine, California, United States) and a quarter waveplate (AQWP3, 25 Boldervision, Boulder, Colorado, United States). The remote objective in our case was chosen to be Olympus 20 × 0.6 NA (MXPLFLN, Shinjuku, Japan). This ensured that the angular aperture of O1 is always higher than the angular aperture of O2 (ASI multi-immersion objective 54-12-8 with NA 0.64 in water) and ${O2}^{\prime }$ (ASI multi-immersion objective 54-10-12 with NA 0.36 in water). A 7-mm-diameter mirror (PF03-03-F01, Thorlabs) is mounted on a linear focus actuator (BLINK^®^, Thorlabs). The reflected light is then collected by the remote objective (O1) and passes through the QWP, where the laser beam changes the polarization to an orthogonal state and transmits through the PBS. The laser beam is then ready to be fed into the relay optics, which comprises three lenses [$L_{P1}$, $L_{P2}$, and $L_{P3}$ (ACT508-300-A, Thorlabs)], and the path compensator with four mirrors and a linear stage.

## 3 Results

We compared the remote focusing performance for a traditional lens-pair-based setup with our method by quantifying the tightness of focus in fluorescein (Sigma-Aldrich F2456, St. Louis, Missouri, United States) over axial ranges. We used two metrics to compare this performance: the full-width-at-half-maximum (FWHM)^18^ and the focal volume.^19^ The FWHM was calculated by fitting a Gaussian to the line profile, and the focal volume was calculated considering the pixel area comprising 40 to 50% of the maximum intensity. The detailed optical paths indicating the intermediate distances between the optical elements for both objectives are individually illustrated in Fig. S3 in the Supplementary Material. For our geometry, the total path length varies by ∼40  mm, and the path compensator is employed to offset this difference, ensuring the maintenance of the 4f geometry in each case without disturbing the other optical elements.

**Fig. 3:**
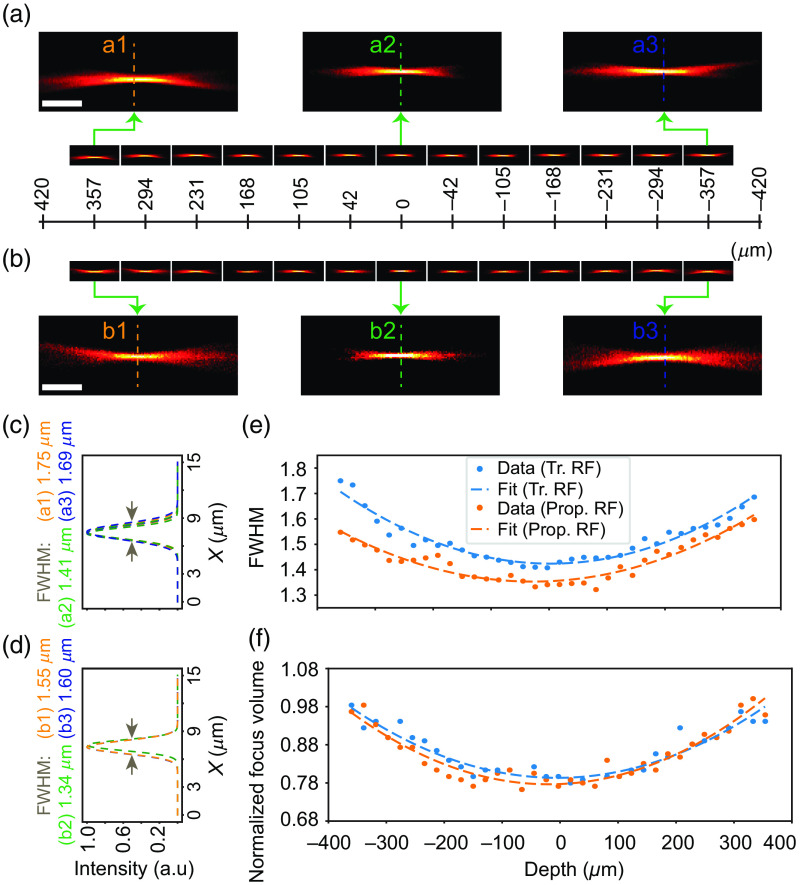
Comparison of the commercial lens-based and the proposed RF technique for 0.36 NA multi-immersion objective in water. (a), (b) The focal volume corresponds to an approximate axial depth of 740  μm (370  μm on either side from the nominal focal plane) for commercial lens-based (a) and the proposed customized (b) RF technique. (c), (d) FWHM corresponding to the dotted lines, a1–a3 from panel (a) and b1–b3 from panel (b), profiles delineate at least similar performance of the proposed customized RF technique (d) over the commercial lens-based RF (c). (e), (f) The FWHM (e) and the normalized focal volume (f) plots prove at least a similar performance, with the added capability of switching objectives while maintaining the core mandates of the RF technique. Scalebars: 12  μm (a), (b). Tr., traditional; prop., proposed.

As evident from Fig. 3, our customized RF approach employing a 0.36 NA water immersion objective, resembles the performance of the traditional lens pair-based RF for the entire axial depth of 740  μm [Figs. 3(a) and 3(b)]. Zoomed-in views of the individual foci [Figs. 3(a) and 3(b)] and their corresponding FWHM plots [Figs. 3(c) and 3(d)] also suggest that our method of carrying our remote focusing results in a comparable quantitative performance. When plotted across an axial range of 740  μm (370  μm on either side of the nominal focal plane), both the FWHM and focal volume calculations depict near-identical performance, demonstrating that our method faithfully recreates the traditional RF setup.

**Fig. 4:**
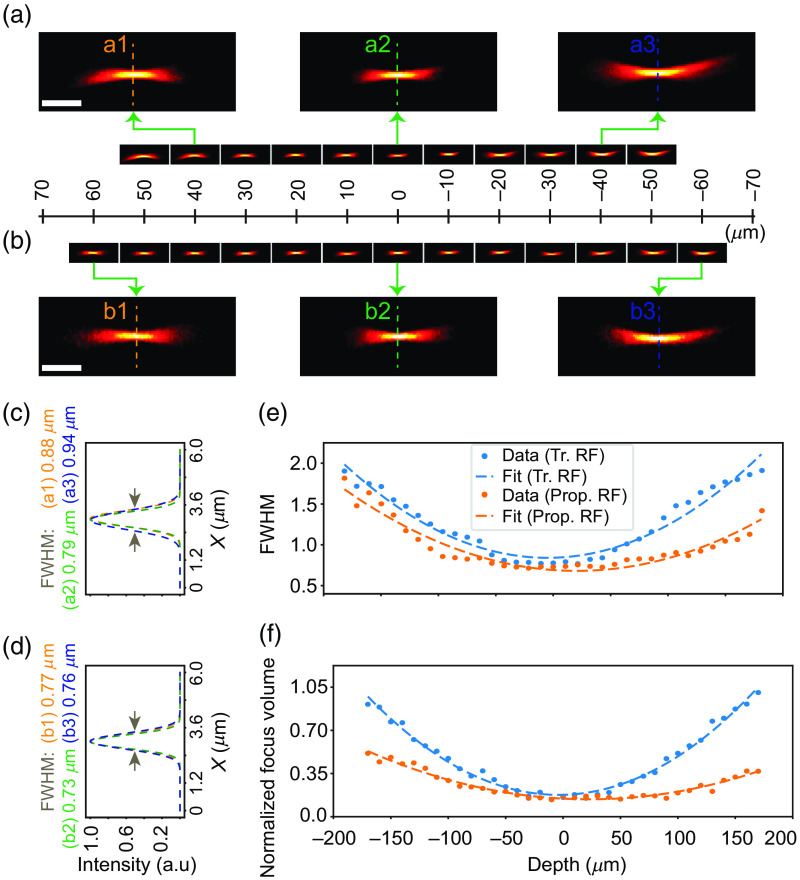
Comparison of the commercial lens-based and the proposed RF technique for a 0.64 NA multi-immersion objective in water. (a), (b) The focal volume corresponds to an approximate axial depth of 120 µm (60 µm on either side from the nominal focal plane) for commercial lens-based (a) and the proposed customized (b) RF technique. (c), (d) FWHM corresponding to the dotted lines, a1-a3 from panel (a) and b1-b3 from panel (b), profiles exhibit similar performance characteristics as found for the 0.36 NA objective. (e), (f) The FWHM (e) and the normalized focal volume (f) plots for both cases demonstrate at least similar, if not better (in the proposed technique), performance characteristics around 350  μm of axial depth. Scale bars: 4  μm (a), (b). Tr., traditional; prop., proposed.

A similar analysis performed utilizing a 0.64 NA (in water) objective depicts interesting observations in Fig. 4. Here, the comparison is shown for an axial depth of ∼120  μm (60  μm on either side) [Figs. 4(a) and 4(b)]. The zoomed-in view and the corresponding FWHM for the dotted line profile (a1 to a3 and b1 to b3 of corresponding color) at three different axial positions demonstrate that our method yields comparable results to the traditional lens pair-based RF [Figs. 4(a)–4(d)]. For the entire 120  μm axial range, the FWHM and the focal volume plots in our customized method closely resemble, if not surpass, the performance of those observed in the traditional lens pair-based RF [Figs. 4(e) and 4(f)].

## 4 Discussion and Conclusion

Depending on the size, shape, and interaction of the objects within the sample user requires different magnification and resolution settings for optimal imaging. Commercial confocal and wide-field microscopes typically feature multiple objectives, allowing users to switch between them to achieve various magnification and resolution levels. The imaging community is actively working on achieving multi-scale modality by switching between various objectives.^11^^,^^20^ Despite the growing popularity of RF techniques in microscopy for volumetric imaging, challenges remain in achieving perfect matching, multi-immersion matching, and multi-resolution imaging. In this letter, we introduced a multiple optical elements-based method that enables the customization of RF. This customized RF approach effectively addresses three key challenges: (a) achieving perfect magnification matching of two objectives immersed in different refractive index (RI) media by creating a lens with a customized focal length and allowing (b) multi-immersion and (c) multi-scale imaging while maintaining magnification matching and 4f geometry without any disruption to the optical equipment. The proposed customized RF technique is both straightforward and potent, offering advantages for achieving a perfect imaging system and facilitating multi-resolution applications.

The selection of the three lenses in the RF system is crucial for accommodating various scales. Users may choose different lens combinations, but transitioning from very low to very high magnification objectives (or vice-versa) may cause significant changes in the beam size. Sometimes such dramatic changes can be challenging to manage with common optical elements, even though the beam size entering the sample objective at the nominal focal plane will match the size of the objective’s back pupil for ideal imaging conditions. With that said, our method can be implemented in both the illumination and detection parts of the microscope, allowing users to customize the system according to their specific requirements. Considering the widespread usage of RF in microscopy, in both illumination and detection schemes, we believe that our method will play a crucial role in multi-scale imaging by adding a new dimension through remote focusing.

## Supplementary Material



## Data Availability

The raw dataset used for this study is available upon request to the corresponding author.
