# Supplementary material for: Remote refocusing for multi-scale imaging
Source: J Biomed Opt. 2024 Aug 8;29(8):080501. doi: 10.1117/1.JBO.29.8.080501 (PMC11309005; doi:10.1117/1.JBO.29.8.080501)
Supplement: Supplementary file 1 [file JBO_029_080501_SD001.pdf]

Remote refocusing for multi-scale imaging

MD NASFUL HUDA PRINCE[1], NIKHIL SAIN[1], TONMOY CHAKRABORTY[1,2,*]

[1]*Department of Physics and Astronomy, University of New Mexico, Albuquerque, NM 87131, USA*
[2]*Comprehensive Cancer Center, University of New Mexico, Albuquerque, NM 87102, USA*
*tchakraborty@unm.edu

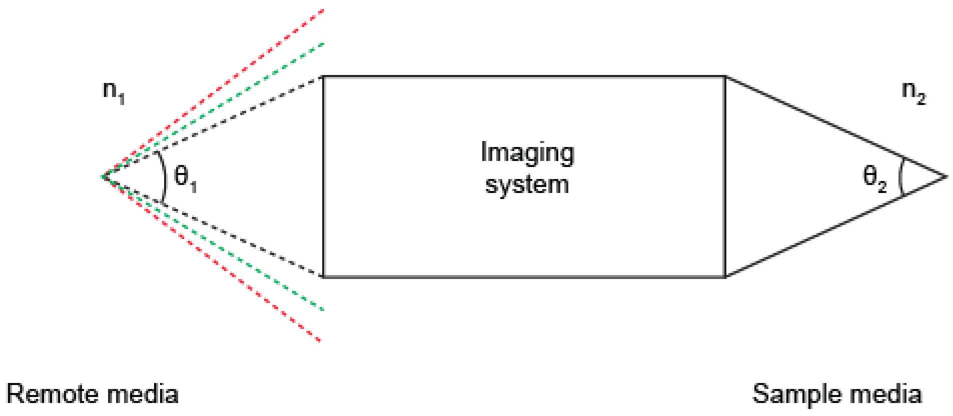

Supplementary Fig. S1 | Criterion to choose remote objective. A remote focusing based imaging system where the perfect imaging system requires the angular-aperture of the remote medium (Θ_1) equal to the angular-aperture of the sample medium (Θ_2). This means that when trying to choose the remote objective one must make sure that the angular aperture should be $\Theta_1 \geq \Theta_{12}$ in order to not lose resolution.

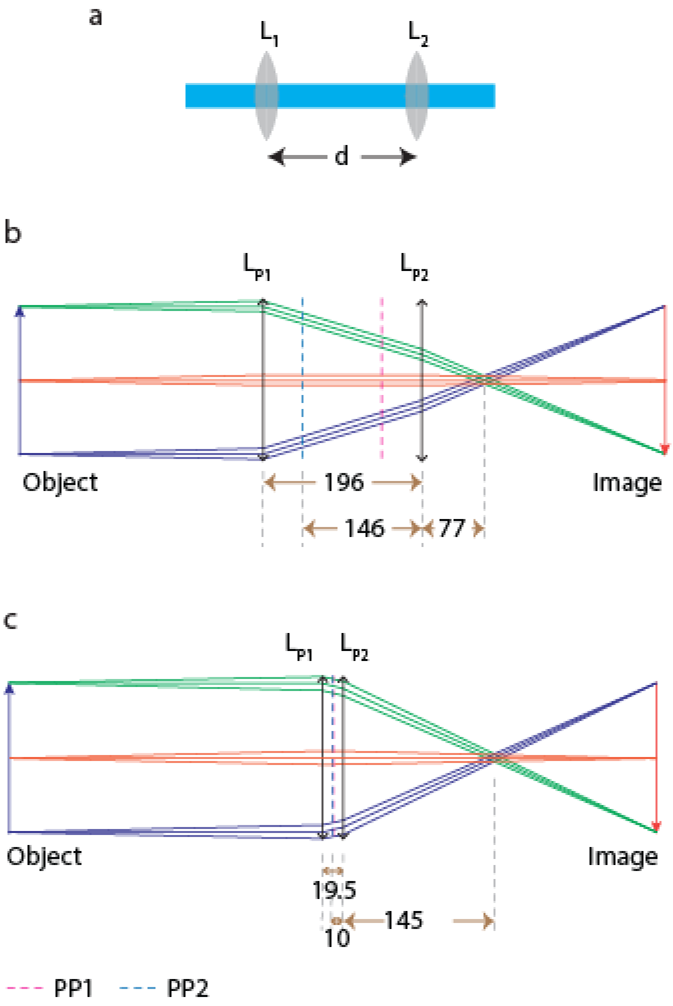

Supplementary Fig. S2 | Two lens system. a, Creation of customized focal length by manipulating the intermediate distance (d) between two lenses (L_1 and L_2). **b-c,** Ray tracing of customized focal length using two identical lenses (L_{P1} and L_{P2}) to match the magnification of 0.64 NA (in water) objective (**b**) and 0.36 NA (in water) objective (**c**). The incoming parallel rays are focused to a distance of customized focal length, measured from the 2nd principal plane of the combined system. The resulting focal length is adjusted by manipulating the intermediate linear distance between the lenses. All distances shown in the figure are measured in millimeters. PP, principal plane.

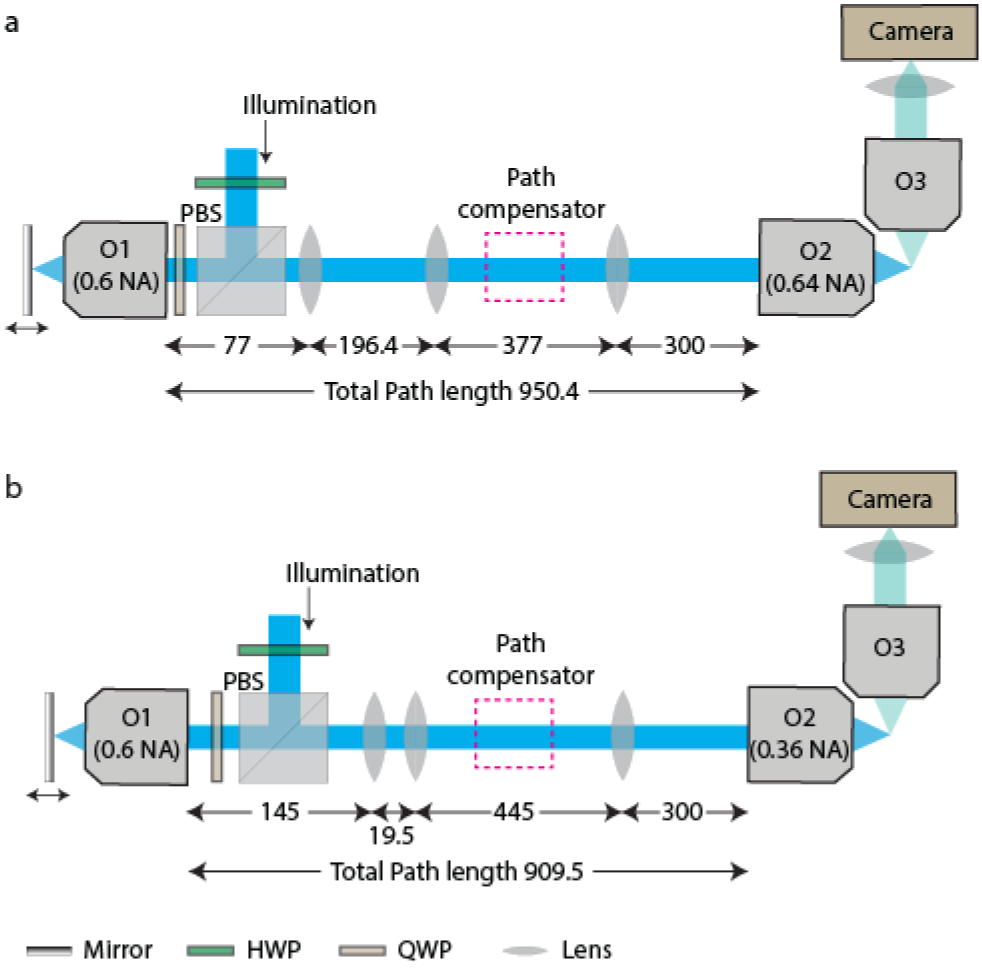

Supplementary Fig. S3 | Optical path for matching two different objectives. a-b, Individual optical path indicating the lengths necessary for a perfect match with 0.64 NA (in water) objective (**a**) and 0.36 NA (in water) objective (**b**), for the same remote objective (O1) using identical optical elements. The sole adjustment needed to switch objectives while preserving magnification matching is to linearly move the position of two lenses and two mirrors. Manipulating the distance between the two lenses creates a customized focal length, and the path compensator (shown as a magenta square box) adjusts the total path length required to maintain the *4f* geometry. All distances shown in the figure are not to scale and are measured in millimeters.

Supplementary Note 1

A lens with a customized focal length (f_{eq}) can be created by combining two commercially available lenses having different focal lengths (f_1 and f_2) (**Supplementary Fig. S3**). The equivalent focal length of the given lens pair can be adjusted by manipulating the intermediate distance between the lens pair as governed by the following equation:

$$\frac{1}{f_{eq}} = \frac{1}{f_1} + \frac{1}{f_2} - \frac{d}{f_1 f_2} \qquad 1$$

The equivalent focal length was measured based on the principal plane of the lens-pair system (**Supplementary Fig. S2b** and **Supplementary Fig. S2c**). We computed cardinal points of the system to determine the lens position required for the resulting equivalent focal length. ABCD matrices for thin lens approximation were employed to compute the cardinal points of the combined lens system [21]:

$$\begin{bmatrix} A & B \\ C & D \end{bmatrix} = \begin{bmatrix} \left(1 - \frac{d}{f_1}\right) & d \\ \left[\frac{1}{f_2}\left(\frac{d}{f_1} - 1\right) - \frac{1}{f_1}\right] & \left(1 - \frac{d}{f_2}\right) \end{bmatrix} \qquad 2$$

Achieving perfect magnification matching necessitates the precise placement of lenses in a correct position. The aforementioned computation yields the exact positions of the lenses required to construct a customized focal length.
